# Identification of combined biomarkers for predicting the risk of osteoporosis using machine learning

**DOI:** 10.18632/aging.204084

**Published:** 2022-05-17

**Authors:** Zhenlong Zheng, Xianglan Zhang, Bong-Kyeong Oh, Ki-Yeol Kim

**Affiliations:** 1 Department of Dermatology, Yanbian University Hospital, Yanji, Jilin Province, China; 2 Department of Dermatology and Cutaneous Biology Research Institute, Severance Hospital, Yonsei University College of Medicine, Seoul, Korea; 3 Department of Pathology, Yanbian University College of Medicine, Yanji, Jilin Province, China; 4 Oral Cancer Research Institute, Yonsei University College of Dentistry, Seoul, Korea; 5 Institute for the Integration of Medicine and Innovative Technology, Hanyang University College of Medicine, Seoul, Korea; 6 BK21 PLUS Project, Department of Dental Education, Yonsei University College of Dentistry, Seoul, Korea

**Keywords:** osteoporosis, risk prediction, gene expression, combined biomarker, machine learning

## Abstract

Osteoporosis is a severe chronic skeletal disorder that affects older individuals, especially postmenopausal women. However, molecular biomarkers for predicting the risk of osteoporosis are not well characterized. The aim of this study was to identify combined biomarkers for predicting the risk of osteoporosis using machine learning methods. We merged three publicly available gene expression datasets (GSE56815, GSE13850, and GSE2208) to obtain expression data for 6354 unique genes in postmenopausal women (45 with high bone mineral density and 45 with low bone mineral density). All machine learning methods were implemented in R, with the GEOquery and limma packages, for dataset download and differentially expressed gene identification, and a nomogram for predicting the risk of osteoporosis was constructed. We detected 378 significant differentially expressed genes using the limma package, representing 15 major biological pathways. The performance of the predictive models based on combined biomarkers (two or three genes) was superior to that of models based on a single gene. The best predictive gene set among two-gene sets included *PLA2G2A* and *WRAP73*. The best predictive gene set among three-gene sets included *LPN1, PFDN6,* and *DOHH*. Overall, we demonstrated the advantages of using combined versus single biomarkers for predicting the risk of osteoporosis. Further, the predictive nomogram constructed using combined biomarkers could be used by clinicians to identify high-risk individuals and in the design of efficient clinical trials to reduce the incidence of osteoporosis.

## INTRODUCTION

As a common health threat, osteoporosis is characterized by reduced bone mineral density (BMD) and bone architecture deterioration, consequently weakening the bones and conferring a higher fracture risk [1]. Currently, osteoporosis is not considered to be only a natural phenomenon occurring in older women, as it occurs throughout all stages of life, regardless of age or sex [2].

Various genetic components and environmental factors may contribute to the pathogenesis of osteoporosis [3, 4]. Preventing low BMD during early menopause is a crucial concern for decreasing the risk of osteoporosis [5]. Age and obesity have been proposed as risk factors of fracture, and clinical nomograms have been constructed including these factors to predict the risk of osteoporosis and fracture [6, 7]. Further, BMD is a significant factor of fracture risk, and is thus widely used in clinical practice as an indicator of osteoporosis [8–10]. However, the detailed pathogenesis of osteoporosis has yet to be elucidated, and there is still no effective therapeutic strategy. Identifying a novel therapeutic target for osteoporosis may help to establish a new therapeutic strategy [8]. Toward this end, microarray gene expression analysis could be used to identify essential targets and related signaling pathways involved in the pathogenesis of osteoporosis [11].

Artificial intelligence (AI) simulates human intelligence using machines, especially computer systems. AI can be used to analyze and improve the predictive performance of models in various research areas. Machine learning (ML), a major branch of AI, has been used in conjunction with bioinformatic functional analysis to identify predictive markers of osteoporosis [12, 13]. Kim et al. [14] and Shim et al. [15] developed machine learning models to accurately identify the risk of osteoporosis in postmenopausal women.

In the current study, we analyzed public gene expression data related to osteoporosis to identify putative combined biomarkers for the prediction of osteoporosis risk. We also performed functional annotation of osteoporosis-related genes to establish a systematic approach to discover new molecular targets for the treatment of osteoporosis. Further, we constructed a nomogram for the prediction of osteoporosis risk in clinical practice, which can be used as an objective guideline for assessing a high risk of osteoporosis. We further anticipate that the identification of individuals at high risk of osteoporosis will allow for the design of more efficient therapeutic trials to ultimately reduce the incidence of osteoporosis.

## RESULTS

**Figure 1:**
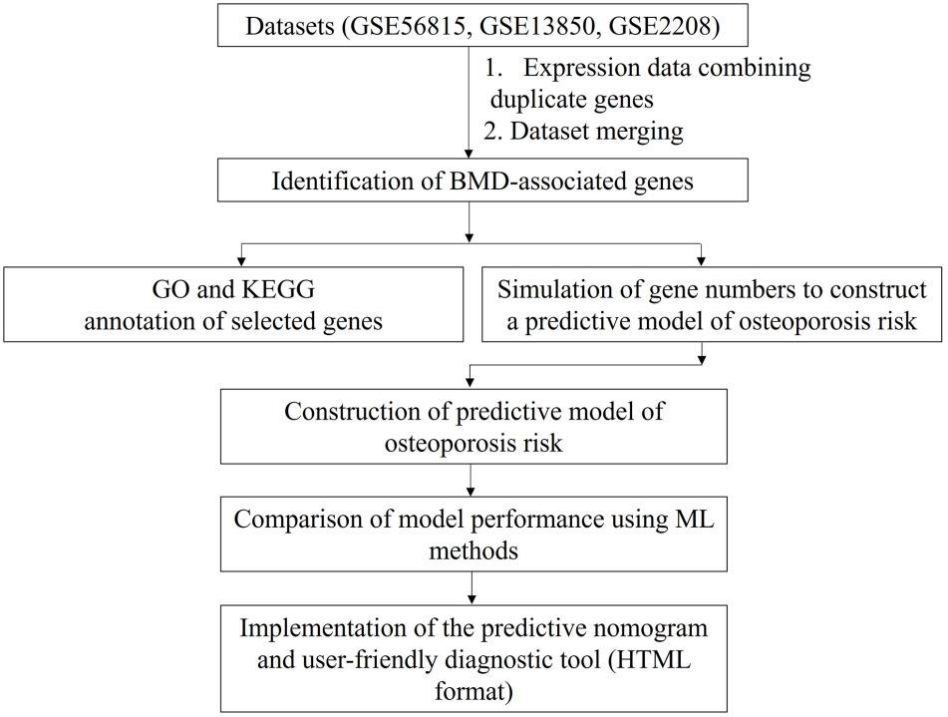
**Study design.** Data for duplicated genes in each gene expression dataset were averaged. The datasets were then merged based on gene name. Finally, osteoporosis-predictive genes were identified, as indicated. BMD: bone mineral density; GO: Gene Ontology; KEGG: Kyoto Encyclopedia Genes Genomes; ML: machine learning; HTML: Hypertext Markup Language format.

We merged three microarray gene expression datasets from postmenopausal women with high and low BMD (GSE56815, GSE13850, and GSE2208) (see Materials and Methods for details). The merged dataset contained gene expression data for 6354 genes from 45 postmenopausal women with high BMD and 45 postmenopausal women with low BMD. We compared the predictive accuracies of the ML algorithms for predicting the risk of osteoporosis using the identified combined biomarkers. The study overview is schematically shown in Figure 1.

### Identification of differentially expressed genes

**Figure 2:**
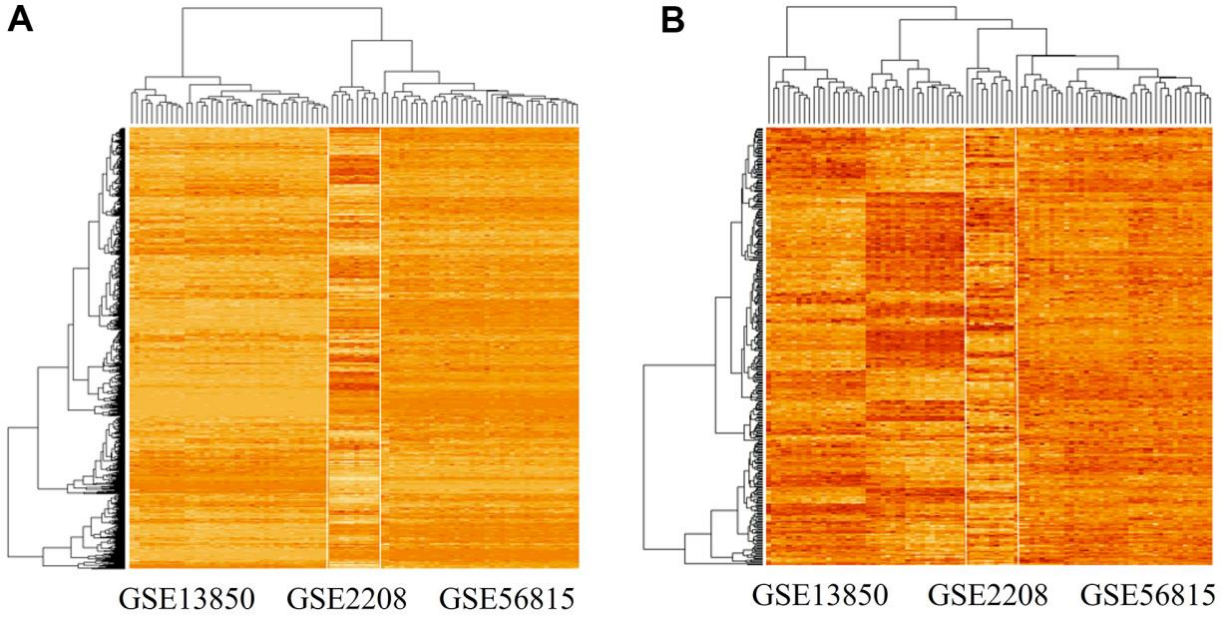
**Gene expression patterns in the three datasets analyzed.** (**A**) Gene expression pattern in the merged microarray dataset, which includes 6354 genes and data from 90 experiments. (**B**) Gene expression pattern of significant differentially expressed genes (n = 378) in high-BMD and low-BMD groups. The genes were identified using the limma package in R; among them, 191 genes were down-regulated and 187 genes were up-regulated.

We used the limma package (see Materials and Methods) to detect 378 differentially expressed genes between the high and low BMD groups. The expression patterns of 6354 genes and the identified 378 differentially expressed genes are shown in Figure 2A, 2B, respectively.

The identified genes showed highly divergent expression patterns between the high and low BMD groups in the GSE13850 dataset, but not in the GSE56815 and GSE2208 datasets (Figure 2B). The upregulated gene set included *TMEM53*, *DRP2*, *ARHGAP44*, *RPS6KA2*, *CEBPE*, *E2F1*, *ASXL2*, *CNTD2*, *GGTLC1*, *SLC22A14*, and *APOE*.

### Gene ontology (GO) analysis of differentially expressed genes

We next performed GO term annotation and pathway enrichment analysis of the differentially expressed genes at the functional level using the Database for Annotation, Visualization and Integrated Discovery (DAVID) (https://david.ncifcrf.gov/) tool. The results are summarized in Table 1.

**Table 1:** Summary of GO terms identified using the DAVID annotation database.

**Category**	**Term**	**Count**	***p*-value^a^**	**Benjamini^b^**
UP_KEYWORDS	Phosphoprotein	239	7.3E-22	2.5E-19
GOTERM_MF_DIRECT	Protein binding	247	2.2E-13	1.2E-10
UP_KEYWORDS	Acetylation	113	3.4E-11	5.8E-9
UP_KEYWORDS	Nucleus	151	9.1E-11	1.0E-8
GOTERM_CC_DIRECT	Nucleoplasm	101	2.0E-10	7.5E-8
GOTERM_CC_DIRECT	Cytoplasm	155	1.0E-9	2.0E-7
UP_KEYWORDS	Alternative splicing	240	1.2E-7	1.1E-5
UP_KEYWORDS	Ubl conjugation	60	7.4E-7	5.0E-5
GOTERM_CC_DIRECT	Nucleus	147	1.7E-6	2.2E-4
UP_KEYWORDS	DNA damage	21	6.4E-6	3.6E-4
UP_KEYWORDS	Methylation	38	2.7E-5	1.3E-3
UP_KEYWORDS	Coiled coil	87	4.2E-5	1.6E-3
UP_KEYWORDS	ATP binding	47	4.3E-5	1.6E-3
UP_KEYWORDS	Isopeptide bond	40	7.4E-5	2.5E-3
UP_KEYWORDS	DNA repair	17	8.5E-5	2.6E-3

^a^*p*-value: modified Fisher’s exact test *p*-value.

^b^Benjamini: Benjamini–Hochberg false discovery rate (FDR)-adjusted *p*-value.

The most significantly enriched GO term was “phosphoprotein” (Table 1). The other significantly enriched terms were protein binding, acetylation, nucleus, nucleoplasm, cytoplasm, alternative splicing, Ubl conjugation, DNA damage, methylation, coiled coil, ATP binding, isopeptide bond, and DNA repair. Each term comprised 17–239 genes.

### Identification of combined predictive markers of osteoporosis risk

**Figure 3:**
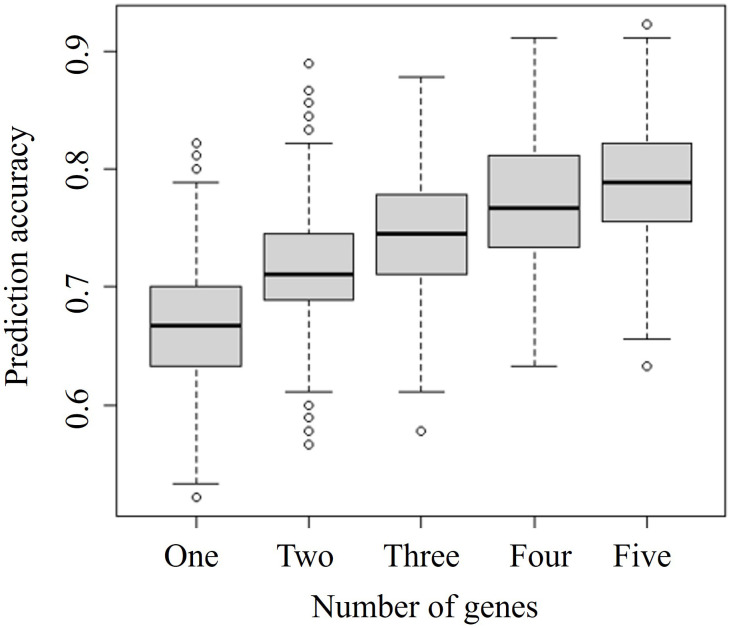
**Comparison of prediction accuracies of combinations of different numbers of genes.** The specific-number gene sets were selected from 378 significant differentially expressed genes identified by the merged microarray dataset using the limma package. Vertical and horizontal axes represent the prediction accuracy and the number of genes considered in combination, respectively.

To select the optimal number of combined biomarkers for risk prediction, we tested random sets of 1–5 genes, with 1000 replicates, and evaluated associations between the number of genes and predictive model accuracy (Figure 3).

The prediction accuracy indicates the probability of concordance between predicted and observed responses. The accuracy increased with an increasing number of combined genes. We focused on identifying a prediction model based on the least number of genes and selected a two-gene set for further analysis. Among the various two-gene sets, the set including *PLA2G2A* and *WRAP73* showed the highest accuracy, at almost 0.9. Ten combined biomarker sets of two or three genes each identified by simulations shown in Figure 3 are listed in Table 2.

**Table 2:** Overview of the 10 sets of combined genes (two or three genes) tested.

**Gene set**	**Genes**	**Description**
1	*PLA2G2A*	Phospholipase A2, membrane associated
	*WRAP73*	Human WD repeat containing, antisense to TP73
2	*DOHH*	Deoxyhypusine hydroxylase
	*SLC22A14*	Solute carrier family 22, member 14
3	*OXTR*	Oxytocin receptor
	*FURIN*	Furin, paired basic amino acid cleaving enzyme
4	*SLC41A3*	Solute carrier family 41, member 3
	*BBIP1*	BBSome interacting protein 1
5	*TBP*	TATA-binding protein
	*TICAM1*	Toll-like receptor adaptor molecule 1
6	*MGRN1*	Mahogunin ring finger 1
	*PDGFB*	Platelet-derived growth factor subunit B
	*ZNF764*	Zinc finger protein 764
7	*PSPC1*	Paraspeckle component 1
	*MPI*	Mannose phosphate isomerase
	*EIF5*	Eukaryotic translation initiation factor 5
8	*WDR6*	WD repeat-containing protein 6
	*PFDN6*	Prefoldin subunit 6
	*PSPC1*	Paraspeckle component 1
9	*ADM2*	Adrenomedullin 2
	*MFSD10*	Major facilitator superfamily domain containing 10
	*PAFAH1B1* (*LIS1*)	Platelet-activating factor acetylhydrolase 1b regulatory subunit 1
10	*LPIN1*	Lipin-1
	*PFDN6*	Prefoldin subunit 6
	*DOHH*	Deoxyhypusine hydroxylase

### Performance comparison of risk predictive models for osteoporosis

We then compared the performance of the risk predictive models for osteoporosis using different ML algorithms. For this experiment, the dataset was randomly split into training (70% of data) and testing (30% of data) datasets. Random dataset spilt was processed repeatedly 100 times, and the model performance is summarized according to mean values and standard deviations calculated for all processing cycles in Table 3.

**Table 3:** Comparison of predictive accuracies of models with training and testing datasets.

		**Training dataset**				**Testing dataset**			
**Single genes**									
		LDA	KNN	SVM	RF	LDA	KNN	SVM	RF
		0.667	0.731	0.740	0.999	0.662	0.650	0.641	0.603
		0.057	0.049	0.055	0.003	0.093	0.095	0.092	0.102
**Combined biomarkers**									
Gene set	Genes	LDA	KNN	SVM	RF	LDA	KNN	SVM	RF
*1*	*PLA2G2A* *WRAP73*	0.893 0.026	0.888 0.035	0.899 0.031	1.000 0.000	0.873 0.077	0.859 0.060	0.864 0.055	0.841 0.088
*2*	*DOHH* *SLC22A14*	0.802 0.026	0.879 0.026	0.882 0.028	1.000 0.000	0.800 0.071	0.852 0.057	0.826 0.070	0.829 0.062
*3*	*OXTR* *FURIN*	0.860 0.027	0.840 0.038	0.879 0.030	1.000 0.000	0.851 0.061	0.783 0.065	0.808 0.061	0.789 0.073
*4*	*SLC41A3* *BBIP1*	0.887 0.028	0.889 0.028	0.935 0.022	1.000 0.000	0.881 0.055	0.838 0.060	0.852 0.055	0.799 0.065
*5*	*TBP* *TICAM1*	0.854 0.041	0.867 0.030	0.881 0.029	1.000 0.000	0.827 0.071	0.815 0.066	0.803 0.070	0.782 0.066
*6*	*MGRN1* *PDGFB* *ZNF764*	0.863 0.025	0.880 0.027	0.894 0.026	1.000 0.000	0.834 0.065	0.827 0.049	0.819 0.067	0.842 0.057
*7*	*PSPC1* *MPI* *EIF5*	0.832 0.046	0.866 0.023	0.889 0.025	1.000 0.000	0.810 0.072	0.854 0.057	0.853 0.060	0.829 0.059
*8*	*WDR6* *PFDN6* *PSPC1*	0.853 0.030	0.856 0.033	0.869 0.027	1.000 0.000	0.843 0.059	0.786 0.069	0.805 0.059	0.798 0.071
*9*	*ADM2* *MFSD10* *PAFAH1B1*	0.834 0.028	0.817 0.034	0.858 0.029	1.000 0.000	0.799 0.070	0.734 0.075	0.771 0.082	0.789 0.060
*10*	*LPIN1* *PFDN6* *DOHH*	0.869 0.036	0.885 0.026	0.927 0.015	1.000 0.000	0.858 0.037	0.860 0.020	0.873 0.048	0.920 0.036

LDA, Linear discriminant analysis; KNN, k-Nearest neighbors; SVM, Support vector machine; RF, Random forest.

ML methods are indicated, and values are the mean (top) and standard deviation (bottom) calculated from 100 reiterations.

We compared the performance of two model types: one predicting the risk probability of osteoporosis based on a single gene and the other predicting risk based on combined biomarkers (two or three genes). The performance of models based on combined biomarkers was superior to that of models based on single genes (Table 3). For single-gene models, the predictive accuracies were 0.667–0.999 with the training dataset and 0.603–0.662 with the testing dataset. For models based on combined biomarkers, random forest (RF) was the best-performing model with the training dataset (accuracy = 1.0). Performances with the test dataset tended to depend on the combined biomarkers used. Although RF exhibited the best performance with the training dataset, its performance with the testing dataset was worse than that of other models. When two genes were considered in a model, the best predictive gene set was *PLA2G2A* and *WRAP73*. When three genes were considered, the best predictive gene set was *LPN1*, *PFDN6*, and *DOHH*.

### Nomogram construction

**Figure 4:**
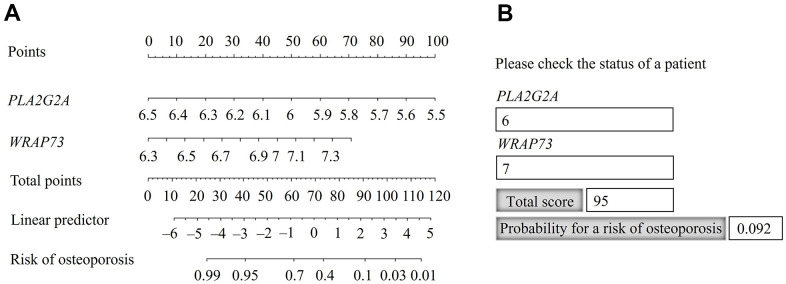
**Nomogram for predicting the probability of osteoporosis risk.** (**A**) Identification of the probability of osteoporosis risk for an individual patient. (**B**) Practical use of the nomogram, available in Hypertext Markup Language (HTML) format.

A nomogram was constructed using the gene set of *PLA2G2A* and *WARP73* (Figure 4A), as the best combination of two genes for predicting the risk of osteoporosis (Table 3).

The risk probability of osteoporosis increased when the calculated point total decreased. For the point total of 95, the risk probability was 9.2% and for the point total of 40, the risk probability was 95% (Figure 4A). For practical application, we constructed the nomogram in Hypertext Markup Language (HTML) format and populated it with the calculated total scores and probabilities (Figure 4B). The calculated point total can also be used for stratification according to the risk probability of osteoporosis.

## DISCUSSION

In this study, we analyzed merged microarray datasets of gene expression in postmenopausal women with high and low BMD. Using ML methods, we identified *PLA2G2A* and *WRAP73* as the optimal combined biomarker set for predicting the risk of osteoporosis, and constructed a related nomogram for practical use. The devised nomogram will help clinicians to identify patients at high risk of osteoporosis, allowing timely treatment or a prevention strategy. Further, the obtained data provide insights into the development of osteoporosis.

In addition to biomarker set identification, the current study sheds light on the molecular processes involved in osteoporosis. We identified 378 genes that are significantly differentially expressed in postmenopausal women with high and low BMD. These include *APOE*, *PRKAA1*, and *MAP3K1*, which were previously reported to be associated with Alzheimer’s disease (AD) [16–18]. As a common degenerative disease, AD and osteoporosis mainly occur in the elderly population [19, 20]. Woodman [21] and Xiong et al. [22] reported that both decreased BMD and fractures are common phenomena in AD patients, and that AD target genes might be risk factors for osteoporosis. These observations coincide with the osteoporosis-related genes identified herein, which could also be AD biomarkers.

Among the combined gene sets identified in this study (Table 2), *PLA2G2A* and *WRAP73* constituted the optimal identified biomarker set. *PLA2G2A* is associated with osteosarcopenia, and *PLA2G2A* overexpression is reported to be a valuable finding for the clinical management of sarcopenia in elderly women with osteoporosis [23, 24]. Further, PLA2G2A influences osteoclastic bone resorption by facilitating the production of prostaglandin, a key modulator of bone remodeling [25, 26]. *WRAP73* encodes a member of the WD repeat protein family that is implicated in many essential biological functions and pathological processes. Specifically, BIG-3, Wdr5, and Wdr8, members of the WD repeat protein family, have been implicated in osteoblast differentiation and osteogenesis *in vivo* [27–30]. We therefore propose that *PLA2G2A* and *WRAP73* may influence the development of osteoporosis by regulating bone remodeling.

Considering the other identified genes, targeting *OXTR*, which encodes a protein that mediates anabolic skeletal recovery [31, 32], has been suggested as a possible therapeutic target for osteoporosis and obesity. Indeed, Tamma et al. [33] reported that high levels of circulating oxytocin can activate osteoclast *OXTR* to prevent bone resorption by mature osteoclasts. Another differentially expressed gene, *FURIN*, was reported as a hub gene in postmenopausal women with low BMD, as indicated by the analysis of regulatory patterns of genes potentially associated with osteoporosis risk uncovered using Bayesian network analysis [34]. *PDGFB*, another gene identified herein, encodes a well-known growth factor required for various crucial biological processes such as embryonic development [35]. In mouse models, bone strength was increased under hematopoietic stem cell-based *PDGFB* therapy [36]. Moreover, as a homodimer of PDGFB, plasma PDGF-BB levels are maintained by estrogen in healthy young women and play a major role in postmenopausal osteoporosis [37]. Furthermore, several studies highlighted *PDGFB* as possible therapeutic target for osteoporosis [38–40]. Finally, Ye et al. [41, 42] reported that *PAFAH1B1* (*LIS1*), another gene identified to be differentially expressed in the current study, can promote osteoclastogenesis via regulating both the differentiation and survival of osteoclast progenitors.

In the current study, phosphorylation was the pathway that was the most significantly enriched in osteoporosis-related genes, according to the GO DAVID annotation. Phosphorylation plays an essential role in bone metabolism in humans. For instance, phosphorylation of extracellular bone matrix proteins has been suggested as a risk factor of bone fragility [43]. Further, osteopontin, one of the key representative phosphoproteins in the bone matrix, is considered an early diagnostic biomarker of osteoporosis in postmenopausal women [44].

Although ML methods easily identify trends and patterns in a dataset, they require massive datasets to train on. Considering the small sample size, the first limitation of the current study is that the dataset used does not represent the entire population of individuals with osteoporosis. A model trained on a random sample of a dataset might have poor generalizability and perform poorly outside of that sample. Indeed, the use of large training and testing sets yields predictions that are more accurate and reliable than those obtained using small datasets [45]. The second limitation of the current study is that the subjects were all postmenopausal women. Osteoporosis and its major complication, osteoporotic fracture, affect both men and women, and cause substantial morbidity and mortality worldwide. Although the risk of osteoporosis is higher for women than for men, men suffer greater morbidity and mortality rates following osteoporotic fractures, especially at an advanced age, than women [46].

We presented the implemented a prediction model in the form of a nomogram, i.e., a graphical representation of a statistical model that indicates the probability of a particular clinical outcome. The nomogram constructed herein can serve as an objective guideline for the assessment of osteoporosis in high-risk individuals. The identification of individuals at high risk of osteoporosis would facilitate the design of efficient clinical trials to reduce the incidence of osteoporosis. The constructed nomogram could be used as a test version. As the next step, predictive model incorporating clinical variables and based on specific gene expression in a large dataset relevant to an aging population should be devised.

## MATERIALS AND METHODS

### Data preparation

Three publicly available gene expression datasets for blood monocytes (GSE56815, GSE13850, and GSE2208) were used in the current study. These datasets are accessible from the public Gene Expression Omnibus (GEO) microarray database (https://www.ncbi.nlm.nih.gov/geo/). GSE56815 and GSE13850 each consist of data for 20 postmenopausal women with high BMD and 20 postmenopausal women with low BMD (for a total of 22,283 probes). GSE2208 includes data for five postmenopausal women with high BMD and five postmenopausal women with low BMD (for a total of 8623 probes). The three datasets were acquired using the same platform, GPL96 Affymetrix GeneChip Human Genome U133 Array Set. The GSE13850 and GSE56815 datasets contain expression data for 13,516 unique genes, and GSE2208 contains expression data for 8623 unique genes. Since these expression datasets contain duplicated gene symbols, mean expression values for duplicated genes in each dataset were combined and used for downstream analysis. The three datasets were merged according to gene name, to obtain a combined dataset for 45 postmenopausal women with high BMD and 45 postmenopausal women with low BMD with a total of 6354 gene symbols. The study design is shown in Figure 1.

### GO functional annotation

The GO [47] project is a model structured to address the molecular function, biological process, and cellular component for individual genes by large-scale gene annotation. DAVID is a comprehensive functional annotation tool to provide a functional interpretation of a large gene set derived from genomic studies by a clustering algorithm [48]. In the current study, the DAVID online tool was used for pathway enrichment analysis of differentially expressed genes.

A modified Fisher’s exact test *p*-value was used in this study. DAVID can be used to examine thousands of gene sets and test multiple hypotheses. DAVID provides a Benjamini–Hochberg false-discovery rate (FDR)-adjusted *p*-value, with a smaller *p*-value indicating more significant enrichment. The *p*-value and Benjamin–Hochberg FDR were both used to determine the significance of term enrichment for each annotation.

### ML methods

The following ML algorithms were used in the current study [49].

### Linear discriminant analysis (LDA)

LDA is a generalization of Fisher’s linear discriminant, which is used to find a linear combination of features that characterize or separate two or more classes of objects or events. The resulting combination might be used as a linear classifier or for dimensionality reduction before classification. LDA is a dimension-reduction technique that is commonly applied to supervised classification problems. It is mainly used to model differences between groups (i.e., separating two or more groups from each other) [50].

### k-Nearest neighbors (KNN) algorithm

The KNN algorithm is one of the simplest techniques used in ML [9], which is used for both classification and regression. The KNN algorithm works by finding the distance between data points based on the Euclidean distance. KNN computes the distance between each data point and the test data and then calculates the probability that the points are similar to the test data, finally classifying the data points based on the shared highest probabilities [51].

### Support vector machine (SVM)

As one of the representative supervised learning models for pattern recognition and data analysis, SVM is mainly used for classification and regression analysis [52]. The objective of the SVM algorithm is to find a hyperplane in N-dimensional space (where N is the number of features) that distinctly classifies data points. Support vectors are data points that are closer to the hyperplane, which influence the position and orientation of the hyperplane. Using these support vectors, the margin of the classifier is maximized. Deleting the support vectors changes the position of the hyperplane.

### RF

RF is an ensemble learning method for classification, regression, and other tasks. It operates by constructing a multitude of decision trees in the training phase. The output is the mode of classes (classification) or mean/average prediction (regression) of individual trees [53]. The RF algorithm can be used to solve both regression and classification problems.

All ML models were implemented using the R programming language, version 4.1.0 (R Foundation for Statistical Computing, Vienna, Austria) [54], including the GEOquery and limma packages for downloading GEO datasets and identification of significant differentially expressed genes. The nomogram for predicting the risk of osteoporosis was created based on the selected genes.

## References

[1] Akkawi I, Zmerly H. Osteoporosis: Current Concepts. Joints. 2018; 6:122–7. 10.1055/s-0038-1660790 30051110 PMC6059859

[2] Kling JM, Clarke BL, Sandhu NP. Osteoporosis prevention, screening, and treatment: a review. J Womens Health (Larchmt). 2014; 23:563–72. 10.1089/jwh.2013.4611 24766381 PMC4089021

[3] Trajanoska K, Rivadeneira F. The genetic architecture of osteoporosis and fracture risk. Bone. 2019; 126:2–10. 10.1016/j.bone.2019.04.005 30980960

[4] Huang QY, Kung AW. Genetics of osteoporosis. Mol Genet Metab. 2006; 88:295–306. 10.1016/j.ymgme.2006.04.009 16762578

[5] Billington EO, Leslie WD, Brown JP, Prior JC, Morin SN, Kovacs CS, Kaiser SM, Lentle BC, Anastassiades T, Towheed T, Kline GA. Simulated effects of early menopausal bone mineral density preservation on long-term fracture risk: a feasibility study. Osteoporos Int. 2021; 32:1313–20. 10.1007/s00198-021-05826-5 33438038

[6] Nguyen ND, Frost SA, Center JR, Eisman JA, Nguyen TV. Development of prognostic nomograms for individualizing 5-year and 10-year fracture risks. Osteoporos Int. 2008; 19:1431–44. 10.1007/s00198-008-0588-0 18324342

[7] Pongchaiyakul C, Panichkul S, Songpatanasilp T, Nguyen TV. A nomogram for predicting osteoporosis risk based on age, weight and quantitative ultrasound measurement. Osteoporos Int. 2007; 18:525–31. 10.1007/s00198-006-0279-7 17216132

[8] Xia B, Li Y, Zhou J, Tian B, Feng L. Identification of potential pathogenic genes associated with osteoporosis. Bone Joint Res. 2017; 6:640–8. 10.1302/2046-3758.612.BJR-2017-0102.R1 29203636 PMC5935809

[9] Liu YZ, Dvornyk V, Lu Y, Shen H, Lappe JM, Recker RR, Deng HW. A novel pathophysiological mechanism for osteoporosis suggested by an *in vivo* gene expression study of circulating monocytes. J Biol Chem. 2005; 280:29011–6. 10.1074/jbc.M501164200 15965235

[10] Kanis JA, Cooper C, Rizzoli R, Reginster JY, and Scientific Advisory Board of the European Society for Clinical and Economic Aspects of Osteoporosis (ESCEO) and the Committees of Scientific Advisors and National Societies of the International Osteoporosis Foundation (IOF). European guidance for the diagnosis and management of osteoporosis in postmenopausal women. Osteoporos Int. 2019; 30:3–44. 10.1007/s00198-018-4704-5 30324412 PMC7026233

[11] Segal E, Shapira M, Regev A, Pe’er D, Botstein D, Koller D, Friedman N. Module networks: identifying regulatory modules and their condition-specific regulators from gene expression data. Nat Genet. 2003; 34:166–76. 10.1038/ng1165 12740579

[12] Lv M, Cui C, Chen P, Li Z. Identification of osteoporosis markers through bioinformatic functional analysis of serum proteome. Medicine (Baltimore). 2020; 99:e22172. 10.1097/MD.0000000000022172 32991410 PMC7523818

[13] Ralston SH. Genetics of osteoporosis. Proc Nutr Soc. 2007; 66:158–65. 10.1017/S002966510700540X 17466098

[14] Kim SK, Yoo TK, Oh E, Kim DW. Osteoporosis risk prediction using machine learning and conventional methods. Annu Int Conf IEEE Eng Med Biol Soc. 2013; 2013:188–91. 10.1109/EMBC.2013.6609469 24109656

[15] Shim JG, Kim DW, Ryu KH, Cho EA, Ahn JH, Kim JI, Lee SH. Application of machine learning approaches for osteoporosis risk prediction in postmenopausal women. Arch Osteoporos. 2020; 15:169. 10.1007/s11657-020-00802-8 33097976

[16] Serrano-Pozo A, Das S, Hyman BT. APOE and Alzheimer’s disease: advances in genetics, pathophysiology, and therapeutic approaches. Lancet Neurol. 2021; 20:68–80. 10.1016/S1474-4422(20)30412-9 33340485 PMC8096522

[17] Muñoz SS, Garner B, Ooi L. Understanding the Role of ApoE Fragments in Alzheimer’s Disease. Neurochem Res. 2019; 44:1297–305. 10.1007/s11064-018-2629-1 30225748

[18] Wang X, Zimmermann HR, Lockhart SN, Craft S, Ma T. Decreased Levels of Blood AMPKα1 but not AMPKα2 Isoform in Patients with Mild Cognitive Impairment and Alzheimer’s Disease: A Pilot Study. J Alzheimers Dis. 2020; 76:217–24. 10.3233/JAD-191189 32444538 PMC8009325

[19] Chen YH, Lo RY. Alzheimer’s disease and osteoporosis. Ci Ji Yi Xue Za Zhi. 2017; 29:138–42. 10.4103/tcmj.tcmj_54_17 28974906 PMC5615992

[20] Dengler-Crish CM, Elefteriou F. Shared mechanisms: osteoporosis and Alzheimer’s disease? Aging (Albany NY). 2019; 11:1317–8. 10.18632/aging.101828 30779704 PMC6428104

[21] Woodman I. Osteoporosis: Linking osteoporosis with Alzheimer disease. Nat Rev Rheumatol. 2013; 9:638. 10.1038/nrrheum.2013.152 24100462

[22] Xia WF, Jung JU, Shun C, Xiong S, Xiong L, Shi XM, Mei L, Xiong WC. Swedish mutant APP suppresses osteoblast differentiation and causes osteoporotic deficit, which are ameliorated by N-acetyl-L-cysteine. J Bone Miner Res. 2013; 28:2122–35. 10.1002/jbmr.1954 23649480 PMC7104794

[23] Kang YJ, Yoo JI, Baek KW. Differential gene expression profile by RNA sequencing study of elderly osteoporotic hip fracture patients with sarcopenia. J Orthop Translat. 2021; 29:10–8. 10.1016/j.jot.2021.04.009 34036042 PMC8138673

[24] Baus-Domínguez M, Gómez-Díaz R, Corcuera-Flores JR, Torres-Lagares D, Ruiz-Villandiego JC, Machuca-Portillo G, Gutiérrez-Pérez JL, Serrera-Figallo MA. Using Genetics in Periodontal Disease to Justify Implant Failure in Down Syndrome Patients. J Clin Med. 2020; 9:2525. 10.3390/jcm9082525 32764374 PMC7464703

[25] Miyaura C, Inada M, Matsumoto C, Ohshiba T, Uozumi N, Shimizu T, Ito A. An essential role of cytosolic phospholipase A2alpha in prostaglandin E2-mediated bone resorption associated with inflammation. J Exp Med. 2003; 197:1303–10. 10.1084/jem.20030015 12743173 PMC2193787

[26] Krieger NS, Bushinsky DA, Frick KK. Cellular mechanisms of bone resorption induced by metabolic acidosis. Semin Dial. 2003; 16:463–6. 10.1046/j.1525-139x.2003.16100.x 14629607

[27] Li D, Roberts R. WD-repeat proteins: structure characteristics, biological function, and their involvement in human diseases. Cell Mol Life Sci. 2001; 58:2085–97. 10.1007/pl00000838 11814058 PMC11337334

[28] Gori F, Demay MB. BIG-3, a novel WD-40 repeat protein, is expressed in the developing growth plate and accelerates chondrocyte differentiation *in vitro*. Endocrinology. 2004; 145:1050–4. 10.1210/en.2003-1314 14657013

[29] Gori F, Friedman L, Demay MB. Wdr5, a novel WD repeat protein, regulates osteoblast and chondrocyte differentiation *in vivo*. J Musculoskelet Neuronal Interact. 2005; 5:338–9. 16340128

[30] Koshizuka Y, Ikegawa S, Sano M, Nakamura K, Nakamura Y. Isolation, characterization, and mapping of the mouse and human WDR8 genes, members of a novel WD-repeat gene family. Genomics. 2001; 72:252–9. 10.1006/geno.2000.6475 11401440

[31] Liu X, Shimono K, Zhu LL, Li J, Peng Y, Imam A, Iqbal J, Moonga S, Colaianni G, Su C, Lu Z, Iwamoto M, Pacifici M, et al. Oxytocin deficiency impairs maternal skeletal remodeling. Biochem Biophys Res Commun. 2009; 388:161–6. 10.1016/j.bbrc.2009.07.148 19653998

[32] Sun L, Lizneva D, Ji Y, Colaianni G, Hadelia E, Gumerova A, Ievleva K, Kuo TC, Korkmaz F, Ryu V, Rahimova A, Gera S, Taneja C, et al. Oxytocin regulates body composition. Proc Natl Acad Sci USA. 2019; 116:26808–15. 10.1073/pnas.1913611116 31843930 PMC6936484

[33] Tamma R, Colaianni G, Zhu LL, DiBenedetto A, Greco G, Montemurro G, Patano N, Strippoli M, Vergari R, Mancini L, Colucci S, Grano M, Faccio R, et al. Oxytocin is an anabolic bone hormone. Proc Natl Acad Sci USA. 2009; 106:7149–54. 10.1073/pnas.0901890106 19369205 PMC2678458

[34] Zhang L, Peng TL, Wang L, Meng XH, Zhu W, Zeng Y, Zhu JQ, Zhou Y, Xiao HM, Deng HW. Network-based Transcriptome-wide Expression Study for Postmenopausal Osteoporosis. J Clin Endocrinol Metab. 2020; 105:2678–91. 10.1210/clinem/dgaa319 32483604 PMC7320836

[35] Van Den Akker NM, Lie-Venema H, Maas S, Eralp I, DeRuiter MC, Poelmann RE, Gittenberger-De Groot AC. Platelet-derived growth factors in the developing avian heart and maturating coronary vasculature. Dev Dyn. 2005; 233:1579–88. 10.1002/dvdy.20476 15973731

[36] Chen W, Baylink DJ, Brier-Jones J, Neises A, Kiroyan JB, Rundle CH, Lau KH, Zhang XB. PDGFB-based stem cell gene therapy increases bone strength in the mouse. Proc Natl Acad Sci USA. 2015; 112:E3893–900. 10.1073/pnas.1501759112 26150503 PMC4517286

[37] Tang L, Xia Z, Luo Z, Long H, Zhu Y, Zhao S. Low plasma PDGF-BB levels are associated with estradiol in postmenopausal osteoporosis: PDGF-BB mediated by estradiol in women. J Int Med Res. 2017; 45:1332–9. 10.1177/0300060517706630 28606019 PMC5625528

[38] Xie H, Cui Z, Wang L, Xia Z, Hu Y, Xian L, Li C, Xie L, Crane J, Wan M, Zhen G, Bian Q, Yu B, et al. PDGF-BB secreted by preosteoclasts induces angiogenesis during coupling with osteogenesis. Nat Med. 2014; 20:1270–8. 10.1038/nm.3668 25282358 PMC4224644

[39] Chen W, Wasnik S, Fu Y, Aranda L, Rundle CH, Lau KW, Baylink DJ, Zhang X. Unique anabolic action of stem cell gene therapy overexpressing PDGFB-DSS6 fusion protein in OVX osteoporosis mouse model. Bone Rep. 2019; 12:100236. 10.1016/j.bonr.2019.100236 31886323 PMC6920713

[40] Huang J, Yin H, Rao SS, Xie PL, Cao X, Rao T, Liu SY, Wang ZX, Cao J, Hu Y, Zhang Y, Luo J, Tan YJ, et al. Harmine enhances type H vessel formation and prevents bone loss in ovariectomized mice. Theranostics. 2018; 8:2435–46. 10.7150/thno.22144 29721090 PMC5928900

[41] Ye S, Fujiwara T, Zhou J, Varughese KI, Zhao H. LIS1 Regulates Osteoclastogenesis through Modulation of M-SCF and RANKL Signaling Pathways and CDC42. Int J Biol Sci. 2016; 12:1488–99. 10.7150/ijbs.15583 27994513 PMC5166490

[42] Ye S, Fowler TW, Pavlos NJ, Ng PY, Liang K, Feng Y, Zheng M, Kurten R, Manolagas SC, Zhao H. LIS1 regulates osteoclast formation and function through its interactions with dynein/dynactin and Plekhm1. PLoS One. 2011; 6:e27285. 10.1371/journal.pone.0027285 22073305 PMC3207863

[43] Sroga GE, Vashishth D. Phosphorylation of Extracellular Bone Matrix Proteins and Its Contribution to Bone Fragility. J Bone Miner Res. 2018; 33:2214–29. 10.1002/jbmr.3552 30001467

[44] Chang IC, Chiang TI, Yeh KT, Lee H, Cheng YW. Increased serum osteopontin is a risk factor for osteoporosis in menopausal women. Osteoporos Int. 2010; 21:1401–9. 10.1007/s00198-009-1107-7 20238102

[45] Figueroa RL, Zeng-Treitler Q, Kandula S, Ngo LH. Predicting sample size required for classification performance. BMC Med Inform Decis Mak. 2012; 12:8. 10.1186/1472-6947-12-8 22336388 PMC3307431

[46] Harvey N, Dennison E, Cooper C. Osteoporosis: impact on health and economics. Nat Rev Rheumatol. 2010; 6:99–105. 10.1038/nrrheum.2009.260 20125177

[47] Ashburner M, Ball CA, Blake JA, Botstein D, Butler H, Cherry JM, Davis AP, Dolinski K, Dwight SS, Eppig JT, Harris MA, Hill DP, Issel-Tarver L, et al. Gene ontology: tool for the unification of biology. The Gene Ontology Consortium. Nat Genet. 2000; 25:25–9. 10.1038/75556 10802651 PMC3037419

[48] Huang DW, Sherman BT, Tan Q, Collins JR, Alvord WG, Roayaei J, Stephens R, Baseler MW, Lane HC, Lempicki RA. The DAVID Gene Functional Classification Tool: a novel biological module-centric algorithm to functionally analyze large gene lists. Genome Biol. 2007; 8:R183. 10.1186/gb-2007-8-9-r183 17784955 PMC2375021

[49] Zhang X, Jang MI, Zheng Z, Gao A, Lin Z, Kim KY. Prediction of Chemosensitivity in Multiple Primary Cancer Patients Using Machine Learning. Anticancer Res. 2021; 41:2419–29. 10.21873/anticanres.15017 33952467

[50] McLachlan GJ. Discriminant Analysis and Statistical Pattern Recognition: Wiley Interscience. 2004.

[51] Altman NS. An introduction to kernel and nearest-neighbor nonparametric regression. The American Statistician. 1992; 46:11. 10.2307/2685209

[52] Vapnik V. The nature of statistical learning theory. New York: pringer-Verlag. 2000. 10.1007/978-1-4757-3264-1

[53] Breiman L. Random Forests. Machine Learning. 2001; 45:28. 10.1023/A:1010933404324

[54] R. The R Project for Statistical Computing R Foundation. http://www.r-project.org/

